# The Composition of the Dispersion Medium Determines the Antibacterial Properties of Copper (II) Oxide Nanoparticles Against *Escherichia coli* Bacteria

**DOI:** 10.3390/nano15060469

**Published:** 2025-03-20

**Authors:** Olga V. Zakharova, Alexander A. Gusev, Peter A. Baranchikov, Svetlana P. Chebotaryova, Svetlana S. Razlivalova, Elina Y. Koiava, Anna A. Kataranova, Gregory V. Grigoriev, Nataliya S. Strekalova, Konstantin V. Krutovsky

**Affiliations:** 1 Scientific and Educational Center for Environmental Science and Biotechnology, Derzhavin Tambov State University, 392020 Tambov, Russia; nanosecurity@mail.ru (A.A.G.); petrovi4-98@yandex.ru (P.A.B.); sweta-chebotarjova@yandex.ru (S.P.C.); razlivalova8@yandex.ru (S.S.R.); e.koiava.e.02@mail.ru (E.Y.K.); akataranova@bk.ru (A.A.K.); bboykick@outlook.com (G.V.G.); kotova-ns@yandex.ru (N.S.S.); 2 Department of Functional Nanosystems and High-Temperature Materials, National University of Science and Technology «MISIS», 119991 Moscow, Russia; 3 Department of Forest Genetics and Forest Tree Breeding, Faculty of Forest Sciences and Forest Ecology, Georg-August University of Göttingen, Büsgenweg 2, 37077 Göttingen, Germany; 4 Laboratory of Population Genetics, N.I. Vavilov Institute of General Genetics, Russian Academy of Sciences, Gubkin Str. 3, 119333 Moscow, Russia; 5 Genome Research and Education Center, Laboratory of Forest Genomics, Department of Genomics and Bioinformatics, Institute of Fundamental Biology and Biotechnology, Siberian Federal University, 660036 Krasnoyarsk, Russia; 6 Scientific and Methodological Center, G.F. Morozov Voronezh State University of Forestry and Technologies, 8 Timiryazeva Str., 394036 Voronezh, Russia

**Keywords:** antibacterial properties, chemical environment, CuO, *Escherichia coli*, nanoparticles, nonlinear effects

## Abstract

Copper (II) oxide nanoparticles (CuO NPs) attract much attention as a promising antimicrobial agent. We studied the antibacterial properties of three types of CuO NPs against *Escherichia coli* bacteria: flake-shaped particles with a diameter of 50–200 nm and a thickness of 10–20 nm (CuO-CD synthesized by chemical deposition), spherical particles with a size of 20–90 nm (CuO-EE obtained by electrical explosion), and rod-shaped particles with a length of 100–200 nm and a diameter of 30 × 70 nm (CuO-CS commercial sample). We tested how the shape, size, and concentration of the NPs, and composition of the dispersion medium affected the properties of the CuO NPs. We prepared dispersions based on distilled water, a 0.9% NaCl solution, and the LB broth by Lennox and used Triton X-100 and sodium dodecyl sulfate (SDS) as stabilizers. The concentration of NPs was 1–100 mg L^−1^. We showed that the dispersion medium composition and stabilizer type had the greatest influence on the antibacterial effects of CuO NPs. We observed the maximum antibacterial effect for all CuO NP types dispersed in water without a stabilizer, as well as in LB broth with the SDS stabilizer. The maximum inhibition of culture growth was observed under the influence of CuO-EE (by 30%) and in the LB broth with the SDS stabilizer (by 1.3–1.8 times depending on the type of particles). In the saline solution, the antibacterial effects were minimal; in some cases, the CuO NPs even promoted bacterial culture growth. SDS increased the antibacterial effects of NPs in broth and saline but decreased them in water. Finally, among the particle types, CuO-CS turned out to be the most bactericidal, which is probably due to their rod-shaped morphology and small diameter. At the same time, the concentration and aggregation effects of CuO NPs in the colloidal systems we studied did not have a linear action on their antibacterial properties. These results can be used in the development of antibacterial coatings and preparations based on CuO NPs to achieve their maximum efficiency, taking into account the expected conditions of their use.

## 1. Introduction

Rising antibiotic resistance poses a serious threat due to the decreased effectiveness of antimicrobials and increased mortality from infectious diseases. According to projections, the number of deaths related to the antibiotic resistance of microorganisms could increase to 10 million per year by 2050 [1]. The 2022 Global Antimicrobial Resistance and Use Surveillance System (GLASS) reports alarming rates of resistance among common bacterial pathogens. For example, the average prevalence of third-generation cephalosporin-resistant *Escherichia coli* across 76 countries is 42%, while methicillin-resistant *Staphylococcus aureus* is 35%. Urinary tract infections caused by *E. coli* are also of great concern; in 2020, one out of five patients infected with *E. coli* had reduced susceptibility to standard antibiotics [2].

In addition, hospital-acquired infections, which arise as a result of ineffective disinfection in a healthcare facility and are secondary to the patient’s initial diagnosis, also raise serious concerns [3]. The prevalence of hospital-acquired infections, associated mainly with bacterial colonization of biomedical surfaces, typically ranges from 4% to 10% (reaching 30% in intensive care units) in Western industrialized countries, making them the sixth leading cause of death [4].

The global action plan on antimicrobial resistance, endorsed by the World Health Organization, sets out five strategic objectives: (1) increase awareness and understanding of antimicrobial resistance, (2) strengthen surveillance and research, (3) reduce the number of infections, (4) optimize the use of antimicrobials, and (5) ensure sustainable investment in combating antimicrobial resistance [2]. Thus, the search for solutions to combat antibiotic resistance and hospital-acquired infections is prompting scientists around the world to search for new alternative antibacterial agents, including those based on nanoparticles (NPs) [5,6]. NPs have a number of favorable distinctive physical and chemical properties, largely due to their increased surface-to-volume ratio [7]. Metal and metal oxide-based NPs have been the subject of considerable research interest over the past few decades due to their availability, surface modification capabilities, and high reactivity [8,9]. Among the most frequently proposed antimicrobial agents are copper (II) oxide NPs (CuO NPs) [10,11,12]. CuO NPs exhibit high antimicrobial activity against pathogenic microorganisms, making them commercially applicable in paints, fabrics, and in hospitals, either as surface treatments or as antimicrobial coatings [13]. In 2008, the United States Environmental Protection Agency (US EPA) recognized copper (Cu) and its alloys as efficient antimicrobial surfaces [14] capable of killing about 99.9% of bacteria in 2 h [15].

For example, it was shown that self-healing polymer coatings based on linseed oil enclosed in a poly (urea-formaldehyde) shell with the addition of CuO exhibited high antibacterial activity against *E. coli* and *S. aureus* due to the release of CuO NPs in damaged areas [16]. The introduction of Cu-containing additives into commercial coating samples made it possible to reduce bacterial contamination with *S. aureus* by 99.9% [17]. The same efficiency was shown by coatings made of nanostructured Cu obtained by dehydrating ultra-thin films of metallic Cu on glass [18]. Overall, research shows that antimicrobial Cu coatings are a new approach to control healthcare-associated infections [19].

The antimicrobial properties of NPs are directly related to their size [20,21,22], shape [23,24,25], surface functionalization [21,26,27], as well as the composition of the medium and the behavior (aggregation and sedimentation) of NPs [28,29,30,31]. Regarding CuO NPs, there are also studies that assessed the effect of the physicochemical parameters of NPs on their antibacterial properties [11,32,33]. In particular, it is known that smaller particles have a higher antimicrobial effect [11,34,35], which is associated with easier penetration through the cell membrane and the disruption of basic cellular processes [20]. In addition, smaller particles can cause higher levels of oxidative stress [36]. The shape of CuO NPs can also significantly affect their antimicrobial properties. For example, spherical CuO NPs showed stronger antibacterial properties against Gram-positive bacteria, while sheet-shaped CuO NPs were more active against Gram-negative bacteria [37]. Rod-shaped CuO NPs, due to the largest contact surface area, were more effective against *E. coli* and *S. aureus* compared to spherical particles [38].

In addition, the antimicrobial properties of NPs can be affected by surface functionalization with various surfactants. They can have their own antimicrobial properties [39,40] and also enhance the action of other antibacterial agents [41]. For example, Triton X-100, a non-ionic surfactant, is commonly used to increase the permeability of cell membranes but has no pronounced antimicrobial properties [42]. A combination of toluidine blue O (TB)-mediated photodynamic therapy with Triton X-100 showed increased antibacterial efficacy against *Enterococcus faecalis* biofilms due to an increase in the proportion of TB monomers, as well as an increase in membrane permeability and wettability [43]. In addition, Triton X-100 can reduce the antibiotic resistance of microorganisms and reduce their pathogenicity by suppressing the ABC transporter, the phosphotransferase system (PTS), and the ATP supply [44]. Silver NPs (Ag NPs) coated with sodium dodecyl sulfate (SDS) showed a stronger antibacterial effect compared with Ag^+^ ions. It is suggested that due to their amphiphilic properties, Ag NPs effectively penetrate the bacterial cell membrane, resulting in the exudation of cellular components [45].

It is important to note that the interaction of NPs and surfactants can occur with the deliberate use of surfactants, for example, to impart stability to colloidal systems or as components of antimicrobial hygiene products. For example, interaction can occur when treating surfaces with coatings containing NPs with detergents. In this regard, it is necessary to study the antimicrobial properties of NPs by taking into account the various possible scenarios of their interaction with the chemical environment.

Another important aspect that must be taken into account when developing antimicrobial agents and coatings using NPs, including CuO, is their safety with respect to humans and the environment.

CuO NPs have been extensively studied for their cytotoxic effects on eukaryotic cells. Recent research, highlighting both their potential toxicity and the factors influencing it, demonstrated that CuO NPs were more toxic than their bulk chemical counterparts to human lung epithelial (A549) cells due to oxidative stress and mitochondrial damage [46]. Karlsson et al. [47] observed significant cytotoxicity of CuO NPs in the same cell line compared to carbon nanotubes. Similar, Siddiqui et al. [48] reported dose-dependent cytotoxicity of CuO NPs in human hepatocellular carcinoma cells (HepG2) with increased reactive oxygen species (ROS) production and DNA damage. There is evidence that the environment surrounding the NPs significantly affects their cytotoxicity. For example, in a serum-containing cell medium, CuO NPs released significantly more Cu ions than in phosphate-buffered saline, which affected the toxicity of CuO NPs to cultured lung cells [49]. Similar examples of the toxicity of CuO NPs on some plants, aquatic organisms, rodents, and the role of the environmental factor in the toxicity of NPs were emphasized in [50]. Thus, when creating effective antibacterial drugs and coatings based on CuO NPs, it is necessary to take into account their possible cytotoxicity and environmental toxicity, which largely depend on the chemical properties of the environment.

Earlier, we found the effect of particle size (50 and 100 nm), dispersion medium (water and saline), and storage time (0.5 and 24 h) of Cu NP colloids on their antibacterial properties [51]. A decrease in the NP size led to a change in the dependence between toxicity and concentration; toxicity peaks appeared at low concentrations. The antibacterial effect of 50 nm Cu NPs suspensions disappeared after 24 h of storage, while there were no such effects for 100 nm Cu NPs suspensions. A suspension of 100 nm Cu NPs at a concentration of 10 mg L^−1^ showed higher toxicity when water was replaced with saline than suspensions of 50 nm Cu NPs. Another study showed that aqueous dispersions of electroexplosive CuO NPs inhibited the growth of *E. coli*, *P. aeruginosa*, *S. aureus*, and *B. cereus*, but the extent of such an effect was highly dependent on the type of strain tested. In addition, the use of highly purified water and alcohol-containing stabilizers during the synthesis of NPs in liquid prevented coagulation of CuO NPs and significantly affected their physicochemical characteristics and, therefore, antibacterial properties [52].

However, the influence of the size and shape of CuO NPs, as well as the chemical nature of the dispersion medium and stabilizer on the antibacterial properties of CuO NPs, taking into account their concentration, has not yet been comprehensively assessed. This was the main objective of the presented study, which uses *E. coli* bacteria as a target bacteria. *E. coli* bacteria is one of the most frequently used and well-studied test object [53], so we considered it relevant for our work.

## 2. Materials and Methods

### 2.1. Nanoparticles

Three types of CuO NPs were used in this study: (1) obtained through chemical precipitation (CuO-CD) [54], (2) obtained via electrical explosion of a conductor in air (Advanced Powder Technologies LLC, Tomsk, Russian Federation) (CuO-EE), and (3) a commercial sample purchased from Sigma-Aldrich (St. Louis, MO, USA) (CuO-CS). The morphology and elemental composition of the NPs were analyzed using a high-resolution Merlin scanning electron microscope (SEM) (Carl Zeiss, Oberkochen, Germany) with energy dispersive X-ray (EDX) analysis using a 10 mm^2^ SDD Detector—X-Act energy dispersive analyzer (Oxford Instruments, Oxford, UK). The phase composition of CuO NPs was investigated via powder X-ray diffraction (XRD) using a D2 Phaser X-ray diffractometer (Bruker AXS, Pforzheim, Germany) with a CuKα anode. The analysis of diffraction patterns was carried out using the PDF-2 database. The calculation of the quantitative ratio of the recorded phases was performed using the Rietveld method.

### 2.2. Dispersed Systems of Nanoparticles

Distilled water (pH 7.0 ± 0.2), the physiological saline solution (NaCl 0.9%, pH 7.0 ± 0.2) (Gematek LLC, Tver, Russian Federation), and the LB broth by Lennox (pH 7.0 ± 0.2) (DIA-M, Moscow, Russian Federation) were used as the dispersion media for the studies. The use of distilled water and saline as media options is explained by the need to explore various realistic scenarios of interactions between bacteria and NPs, including those in which biochemical and osmotic conditions will not be optimal for bacteria. The dispersion media were poured into 100 mL glass beakers; then, NP samples (100 mg) were added there, stirred with a glass rod, and processed in an ultrasonic bath Elmasonic S15H (Elma Schmidbauer GmbH, Singen, Germany). The operating frequency was 37 kHz, and the power was 60 W. The treatment was carried out for 15 min in the 3 × 5 min mode with a minute break between approaches (to reduce the suspension temperature, since the long-term treatment heats up the suspension and causes the particles to aggregate). From the obtained suspensions (1 g L^−1^), suspensions with a concentration of 100 and 10 mg L^−1^ were prepared via dilution, which were then used to study the antibacterial effects. In addition, we assessed the effect of stabilizers in the composition of NP dispersions on their antibacterial properties. The nonionic surfactant Triton X-100 at 100% solution (pre-diluted with distilled water to 10%) (Suzhou Yacoo Science Co., Ltd., Suzhou, Jiangsu, China) and ionic SDS at 10% solution (Sigma-Aldrich, St. Louis, MO, USA) were used as stabilizers. Among anionic surfactants, SDS is the most widely studied and used in pharmaceuticals, household chemicals, and cosmetics. In the development of antimicrobial coatings, hygiene products, etc., the use of SDS can enhance the antimicrobial activity of CuO NPs. To prepare dispersions, 0.0005% surfactant (50 µL of 10% aqueous solution) was added to 1000 mL of dispersion media; then, suspensions of NPs were prepared similarly to dispersions without the surfactant. Thus, the following variants of dispersed systems were tested in this study:

(1) CuO-CD in water, in the physiological saline solution, and in LB broth;
(2) CuO-CD in water, in the physiological saline solution, and in LB broth + Triton X-100;
(3) CuO-CD in water, in the physiological saline solution, and in LB broth + SDS;
(4) CuO-EE in water, in the physiological saline solution, and in LB broth;
(5) CuO-EE in water, in the physiological saline solution, and in LB broth + Triton X-100;
(6) CuO-EE in water, in the physiological saline solution, and in LB broth + SDS;
(7) CuO-CS in water, in the physiological saline solution, and in LB broth;
(8) CuO-CS in water, in the physiological saline solution, and in LB broth + Triton X-100;
(9) CuO-CS in water, in the physiological saline solution, and in LB broth + SDS.

The analysis of the behavior of NPs in dispersions was carried out on solutions with the maximum particle concentration (100 mg L^−1^), and the solutions were then used in further biological tests. To determine the aggregation of particles and the stability of dispersed systems, the size distribution of NPs and the ζ-potential were estimated using a high-performance two-angle particle and molecular size analyzer Zetasizer Nano ZS (Malvern Instruments Ltd., Malvern, UK).

### 2.3. Microbiological Analysis

**Figure 1:**
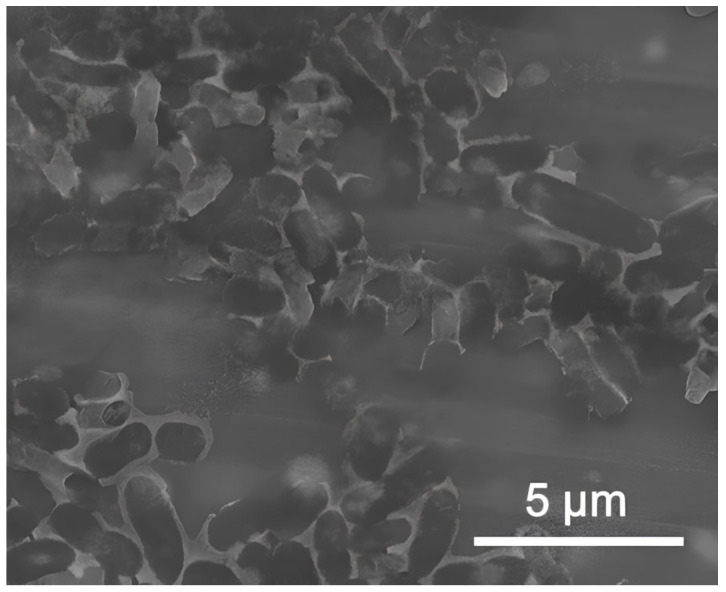
Scanning electron microscope (SEM) image of the studied *E. coli* bacteria (no exposure).

The luminescent strain *E. coli* K12 TG1 [55,56,57] (Figure 1) was used as the test bacteria, as it constitutively expressed the *luxCDABE* genes of the natural marine microorganism 54D10, which was produced by the Immunotech company under the commercial name «Ecolum» (Immunotech, Moscow, Russia) [58,59,60,61,62,63].

Bacteria modified with *luxCDABE* genes were used for rapid preliminary photoluminescence assessments of CuO NP toxicity, in particular for the subsequent selection of the effective concentrations in experimental groups. Then, all subsequent experiments were also carried out with these bacteria according to the preliminary results. The choice was due to the fact that among reporter genes, the lux reporter is distinguished by the fact that light can be measured online without disruption of bacterial cells using optical devices. The use of five *luxCDABE* reporter genes allows for continuous light production without adding exogenous compounds. Additional advantages of the lux reporter system include very low background noise, high sensitivity, and a wide dynamic range. Monitoring changes in bioluminescence intensity provides a direct assessment of the effect of a physical or chemical agent on microbial metabolism [64,65]. The culture was reconstituted according to the manufacturer’s recommendations. Namely, the vial with the lyophilized biosensor was opened, and 10 mL of distilled water (room temperature, pH 6.8–7.4) was added and left for 1 h, thus obtaining the original suspension of the biopreparation. The reconstituted suspension was centrifuged for 5 min at 5000 rpm; the supernatant was removed, and fresh LB nutrient medium according to Lennox was added. The cells were incubated at 37 °C on a Multitron rotary shaker (Infors HT, Bottmingen, Switzerland) at 160 rpm until the stationary phase was reached, with an optical density (OD) at 600 nm (OD600) of ~1.1–1.2. The OD of the samples was determined spectrophotometrically in 96-well plates with a volume of 200 μL on a Multiskan Sky spectrophotometer (Thermo Scientific, Waltham, MA, USA). The results were monitored using a bioluminescent technique [50,60,66,67] and a Biotox-10 device luminometer (Nera S, Moscow, Russia). The methodology and results of the analysis are presented in Supplementary Materials.

### 2.4. Bactericidal Test

We centrifuged a 15 mL suspension of bacterial cells for 5 min at 5000 rpm, removed the supernatant, and added one of the media variants. The OD600 value for this strain was brought to 0.1 for further experiments; then, 200 μL aliquots were added to the wells of a 96-well plate, and 22 μL of dispersions containing the desired concentrations of NPs, stabilizers, and media (control) were added to the wells. Cultivation was carried out for 12 h at 37 °C in 96-well plates in a Multiskan Sky spectrophotometer (Thermo Scientific, Waltham, MA, USA). Background OD values (OD of culture media and NP dispersions) were also measured and taken into account when processing the results.

### 2.5. Statistical Analysis

All trials were conducted using three independent biological replicates with three technical replicates (depending on the analysis), which were then analyzed for significant differences (*p* < 0.05) against the control (zero NP concentration) using a one-way analysis of variance test (ANOVA).

## 3. Results

### 3.1. Analysis of Nanoparticles and Their Dispersions

**Figure 2:**
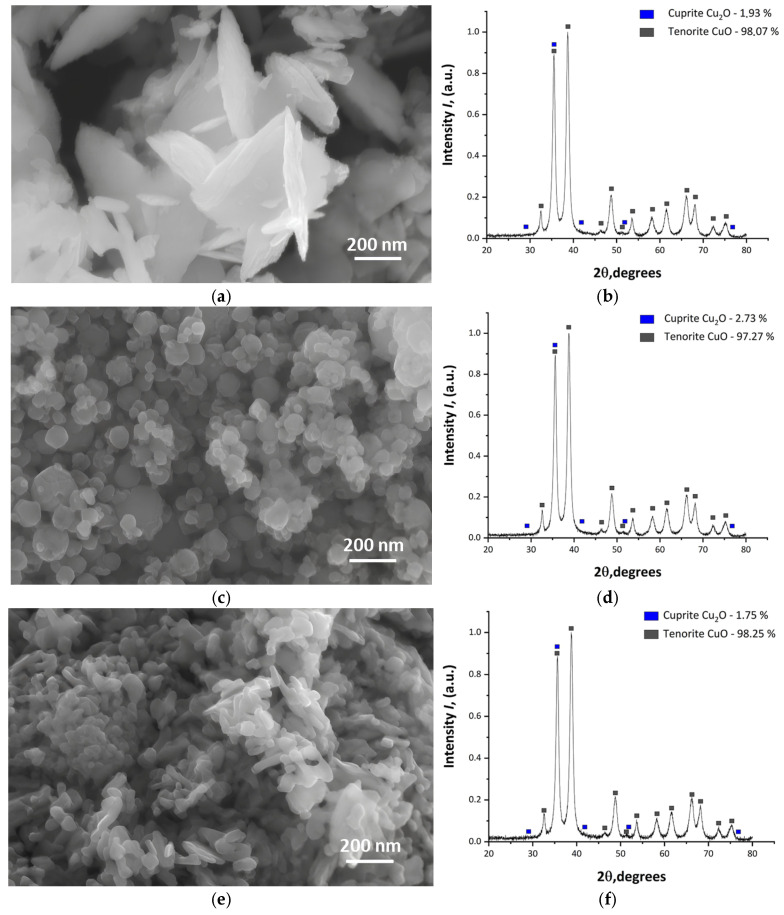
SEM images and the XRD pattern of nanoparticle samples: (**a**,**b**) CuO-CD; (**c**,**d**) CuO-EE; (**e**,**f**) CuO-CS.

SEM of the CuO NPs powder obtained through chemical precipitation (CuO-CD) showed that the powder consists of flake-shaped particles combined into aggregates (Figure 2a). The size of individual particles ranged from 50 to 200 nm in diameter with a thickness of 10–20 nm. The CuO powder obtained using the electric explosion method, CuO-EE, consisted mainly of spherical particles with a diameter of 20–90 nm (Figure 2c). SEM of a commercial sample showed that the NPs in the CuO-CS powder have a rod-shaped morphology with a length of 100–200 nm and a diameter of 30 × 70 nm (Figure 2e). In addition, as can be seen from the micrographs, the particles are highly aggregated. Figure 2b,d,f show the XRD pattern of the analyzed CuO NPs. The sharpness of the X-ray diffraction peaks of CuO indicates that the NPs are crystalline. No additional impurity peaks were detected. Monoclinic cuprite (Cu_2_O) was detected in small amounts.

Visually, all three samples were highly dispersed black powders. EDX analysis showed the presence of Cu and oxygen in all samples.

**Figure 3:**
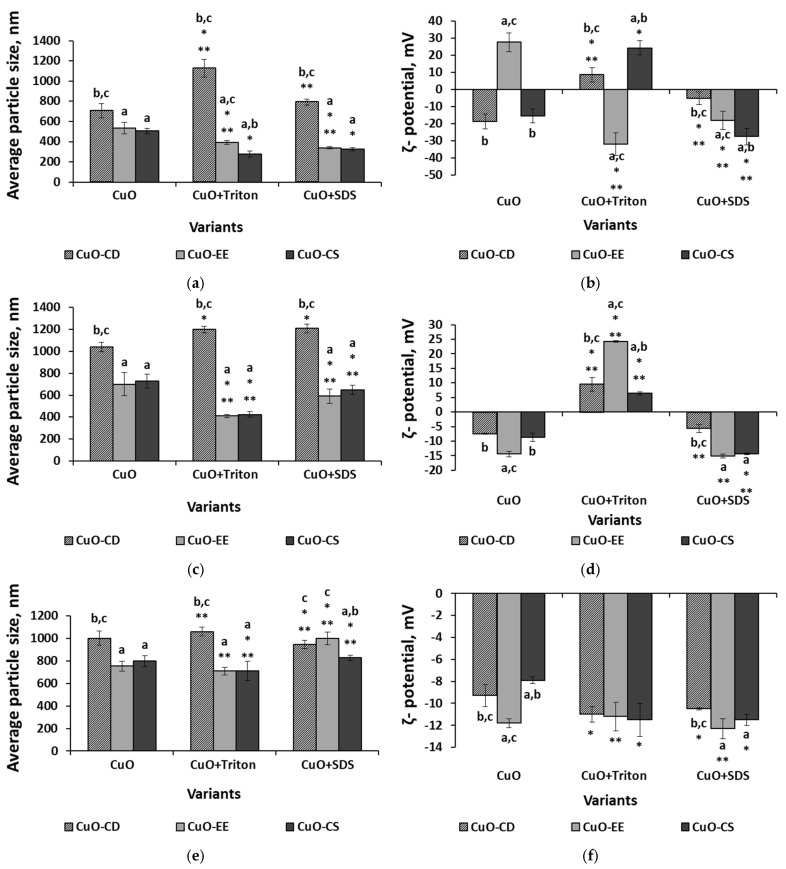
Average particle size and ζ-potential in various media: (**a**,**b**) distilled water, (**c**,**d**) physiological saline solution, and (**e**,**f**) LB broth. Letters above the columns depict a significant difference at *p* < 0.05 with the CuO-CD variant (“a”), the CuO-EE variant (“b”), and the CuO-CS variant (“c”). * Significant differences with CuO without a stabilizer (*p* < 0.05); ** significant differences between variants with the same particle type and different stabilizers (*p* < 0.05).

The use of Triton X-100 made it possible to obtain CuO-EE and CuO-CS dispersions with a smaller particle size and a higher ζ-potential, regardless of the type of medium. At the same time, the opposite trend was observed for CuO-CD in most cases; that is, the addition of Triton X-100 increased the size of aggregates and decreased the ζ-potential. It is also worth noting that in an aqueous medium and in a physiological saline solution medium, the introduction of Triton X-100 changed the charge of particles to the opposite (Figure 3b,d).

The addition of SDS to the dispersion medium had a multidirectional effect on the behavior of NPs in dispersed systems. In the case of CuO-CD, aggregates increased and the ζ-potential decreased under distilled water and physiological saline solution conditions (Figure 3a–d), while under LB broth conditions, SDS had no significant effect (Figure 3e,f). For CuO-EE NPs, a decrease in the aggregate size was observed in water and the physiological saline solution under the influence of SDS, while under LB broth conditions, the average diameter, on the contrary, increased. The ζ-potential index in an aqueous medium decreased with a change in charge upon the introduction of SDS, while in other environments, it remained unchanged. SDS had the most significant effect on CuO-CD in an aqueous medium, where an increase in the average hydrodynamic diameter and a decrease in the particle charge were noted. In a physiological saline solution medium, with an increase in the CuO-CD particle size, the ζ-potential index did not change, while in the LB broth medium, SDS did not affect the behavior of the particles.

### 3.2. Effect of Dispersed Systems of CuO NPs at a Concentration of 100 mg L^−1^ on Bacteria

**Figure 4:**
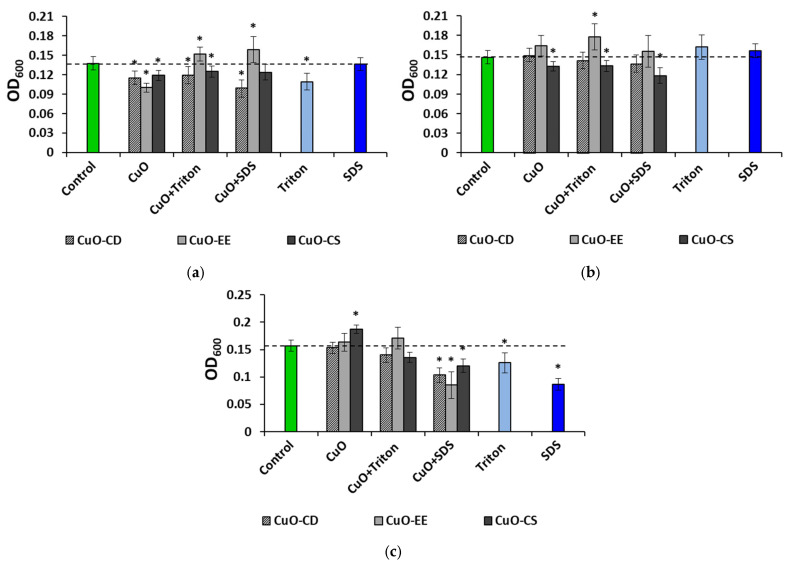
Effect of 100 mg L^−1^ CuO NPs on *E. coli* bacteria in different media: (**a**) distilled water, (**b**) physiological saline solution, and (**c**) LB broth. * Significant differences with the control (*p* < 0.05).

Since the studies of particle sizes in colloids and their ζ-potential were carried out at a concentration of 100 mg L^−1^, we primarily investigated the antibacterial properties of these colloidal systems. It turned out that the dispersion medium has a significant effect not only on the behavior of particles in solutions but also on their antibacterial effect. Thus, it was found that the greatest suppression of bacteria after 12 h of exposure occurred in an aqueous medium without stabilizers, where a decrease in the OD600 indicator was observed for all tested NPs (Figure 4a, Tables S1–S3, and Figure S1).

The lowest indicator was noted for the CuO-EE variant—30% lower than the control values. It is worth noting that the highest positive ζ-potential indicator was noted for this dispersion variant. As mentioned above, the addition of Triton X-100 and SDS changed the charge of CuO-EE in the solution to negative, thereby removing the antibacterial effect of these NPs. It is noteworthy that the average size of aggregates decreased in the following order: CuO-EE > CuO-EE + Triton X-100 > CuO-EE + SDS. The stimulating effect increased. In the case of CuO-CD and CuO-CS, the inhibition of culture growth was observed regardless of the stabilizers. As for the surfactants used, Triton X-100 had a negative effect, while SDS did not have a significant effect. Replacing water with the physiological saline solution reduced the negative effect of NPs on bacteria (Figure 4b). Dispersions based on CuO-CD did not have a significant effect on the analyzed indicator, and CuO-CS slightly reduced OD600, with its maximum in a medium with SDS. Interesting results were obtained for CuO-EE. Despite the fairly high positive charge (24.5 mV) of the particles in the Triton X-100 medium, this dispersion did not have a negative effect on the microorganisms, and moreover, in this case, a slight stimulation (+22%) of culture growth was observed. The stabilizers used did not have an effect in the physiological saline solution. In the LB broth medium, dispersions without a stabilizer and dispersions with Triton X-100 did not have a toxic effect on bacteria, while replacing the stabilizer with SDS provoked a decrease in culture growth by 1.5 times when exposed to CuO-CD, 1.8 times when adding CuO-EE, and 1.3 times in the case of CuO-CS (Figure 4c). It is worth noting that in LB broth, both surfactants had a toxic effect on microorganisms.

It is interesting that a relationship between the size of the aggregates was not observed in solutions, while some relationships between the toxicity of NPs and the ζ-potential were identified in aqueous dispersions. It is noteworthy that the results obtained show the key influence of the dispersion medium and the stabilizer on the antibacterial effect but not the initial size of the particles and their hydrodynamic diameter reported in many other similar studies [68,69,70,71].

### 3.3. Concentration Effects of CuO NPs

**Figure 5:**
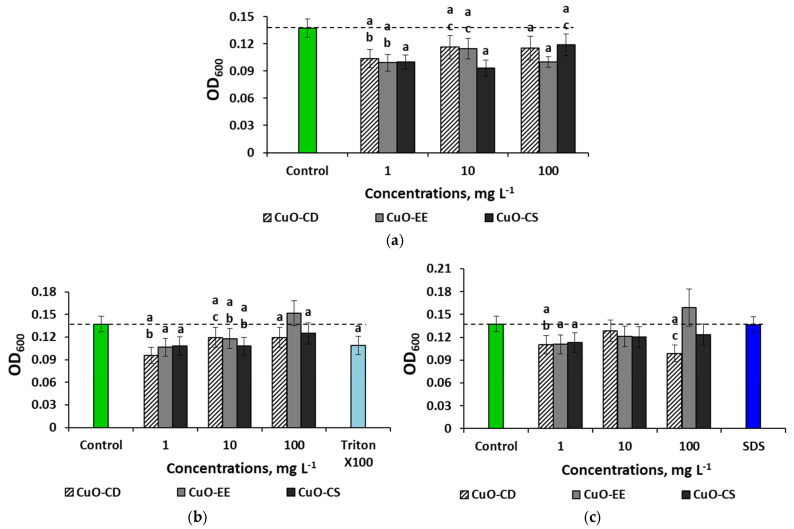
Effects of the CuO NP concentration on *E. coli* bacteria in different environments: (**a**) distilled water, (**b**) distilled water with Triton X-100, and (**c**) distilled water with SDS. Letters above the columns denote statistically significant differences with the control (“a”) and with higher (“b”) or lower (“c”) concentrations (*p* < 0.05).

In addition to assessing the influence of the parameters of colloidal systems on the antibacterial action of NPs in various environments in the presence of various stabilizers, we assessed the effects of the NP concentration. An evaluation of the effect of aqueous dispersions of NPs on bacteria showed a multidirectional effect of CuO NPs and also revealed nonlinear effects of concentration on toxicity (Figure 5).

All aqueous dispersions of CuO obtained without the use of surfactants had a toxic effect on the bacteria (Figure 5a). For CuO-CD, the maximum suppression of bacteria was noted at 1 mg L^−1^, a change of 24% relative to the control. In the case of CuO-EE, the minimum OD values (−27%) were observed at 1 and 100 mg L^−1^, and for CuO-CS, the minimum was noted at 10 mg L^−1^; the OD values decreased by 34% compared to the control values. When using Triton X-100 as a stabilizer, dispersions with the minimum concentration of NPs had the highest toxicity (Figure 5b), while the strongest antibacterial effect was recorded when exposing microorganisms to CuO-CD, where the OD indicator decreased by 1.5 times relative to the control values. In general, the experiments showed an “inverse” dose-dependent effect; that is, the toxicity of NPs decreased with increasing concentration. Replacing the stabilizer with SDS slightly changed the dependence of OD on the type of dispersion (Figure 5c). While maintaining the inhibition of culture growth at a minimum concentration (by 1.2 times), there was no negative effect of the average concentration for all types of particles, and even the maximum dose did not affect the bacteria for CuO-EE and CuO-CS. In the case of CuO-CD, the OD values decreased by 1.4 times at 100 mg L^−1^. This study showed a significant effect of the type of NPs and stabilizers on the toxicity of CuO towards *E. coli* in an aquatic environment. The results also showed the absence of a dose-dependent effect; that is, an increase in concentration did not entail an increase in toxicity. CuO-EE NPs with minimal initial sizes had the lowest toxicity.

**Figure 6:**
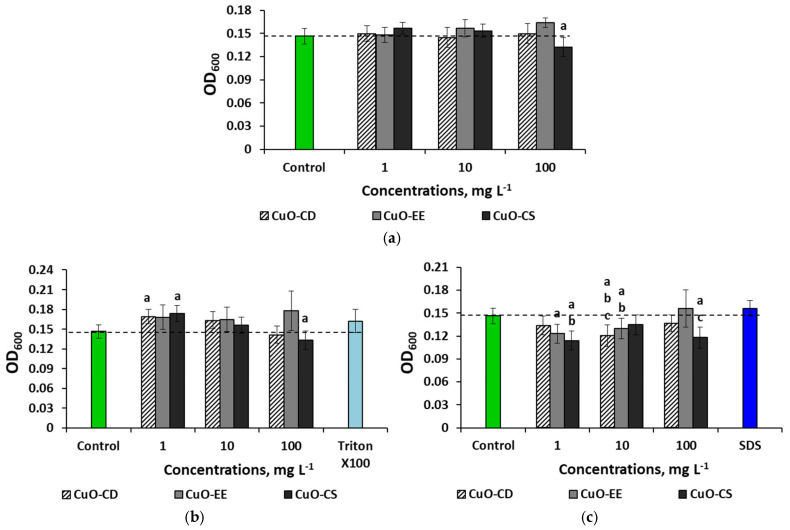
Effects of the CuO NP concentration on *E. coli* bacteria in different environments: (**a**) physiological saline solution, (**b**) physiological saline solution with Triton X-100, and (**c**) physiological saline solution with SDS. Letters above the columns denote statistically significant differences with the control (“a”) and with higher (“b”) or lower (“c”) concentrations (*p* < 0.05).

In the physiological saline solution medium, the NPs did not have a significant effect on bacteria (Figure 6a), with the exception of the CuO-CD variant at a concentration of 100 mg L^−1^, where a slight inhibition of culture growth was observed. When cultivating microorganisms with dispersions prepared using Triton X-100, a negative effect was also noted in the CuO-CD variant at a concentration of 100 mg L^−1^ (Figure 6b).

The addition of CuO-CD and CuO-CS NPs to the medium at a minimum concentration stimulated bacterial culture growth, and the OD values increased by 1.2 times. The use of SDS as a stabilizer reduced bacterial cell growth by 16 and 22% when cultured with 1 mg L^−1^ CuO-EE and CuO-CS, respectively (Figure 6c). The indicators also decreased by 16 and 8% when 1 mg L^−1^ CuO-EE and CuO-CS were added to the medium, respectively. At the maximum concentration, only CuO-CS had a bactericidal effect.

In the case of using the physiological saline solution as a dispersion medium, the bactericidal effect of NPs was noted only for solutions prepared with SDS, as well as dispersions of commercial CuO-CS NPs at the maximum concentration.

**Figure 7:**
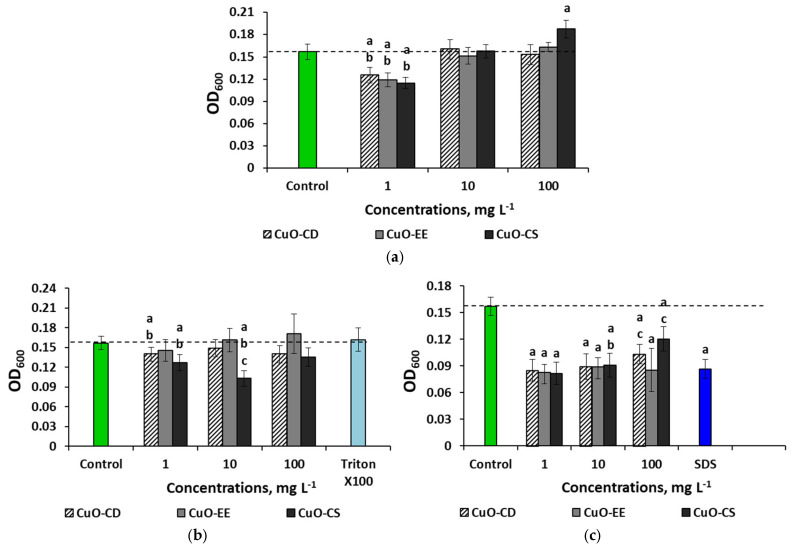
Effects of the CuO NP concentration on *E. coli* bacteria in different environments: (**a**) LB broth, (**b**) LB broth with Triton X-100, and (**c**) LB broth with SDS. Letters above the columns denote statistically significant differences with the control (“a”) and with higher (“b”) or lower (“c”) concentrations (*p* < 0.05).

When culturing bacteria with CuO NPs in LB broth without stabilizers, growth inhibition was noted for all variants of NPs at 1 mg L^−1^; the minimum OD values were recorded when adding CuO-CS to the medium (−27%) (Figure 7a). It is noteworthy that increasing the concentration of CuO-CS to 10 mg L^−1^ leveled out the bactericidal effect of NPs, and at 100 mg L^−1^, as mentioned above, a stimulating effect was noted. CuO-CD and CuO-EE did not have a reliable effect on the bacteria at 10 and 100 mg L^−1^. CuO-CD and CuO-CS dispersions obtained using Triton X-100 inhibited cell growth at 1 mg L^−1^ by 12.5 and 19%, respectively (Figure 7b). Also, a 34% decrease in OD was observed when culturing *E. coli* with 10 mg L^−1^ CuO-CS. As in the case of the physiological saline solution, in the LB broth medium, the most bactericidal CuO dispersions were obtained using SDS (Figure 7c). The lowest OD values (an average 1.8-fold decrease relative to the control) were noted for all types of NPs at 1 and 10 mg L^−1^.

In the LB broth medium, all types of NPs had the maximum toxicity towards *E. coli* at the minimum concentration in the medium without stabilizers, as well as at doses of 1 and 10 mg L^−1^ in the medium with SDS.

In general, the study showed that in the physiological saline solution medium, the tested CuO NPs had no negative effect on bacteria, with the exception of a slight inhibition of culture growth when using dispersions with SDS. In an aqueous medium and the LB broth medium, all types of particles inhibited culture growth at a minimum concentration; however, in water, a concentration of 1 mg L^−1^ inhibited cell growth almost equally, regardless of the surfactant, and in the LB broth medium, the minimum OD values were recorded when using SDS.

As can be seen from the presented data, the dispersion medium and the stabilizers used had the greatest influence on the antibacterial effects of the analyzed NPs. The absence of a linear dose-dependent antibacterial effect was noted for most dispersion variants.

## 4. Discussion

### 4.1. Dispersion Medium Effects

Our study has shown that the dispersion medium has the greatest impact on the behavior of CuO NPs in solutions, as well as on their antibacterial action. The analysis of dispersions prepared without the use of stabilizers showed an increase in the average aggregate size and a decrease in ζ-potential in the following order: water < physiologicalsaline solution < LB broth for electroexplosive particles CuO-EE and commercial CuO-CS, while for CuO-CD, no significant difference was found between the physiological saline solution and LB broth. It is worth mentioning that in all cases, the greatest difference between the aggregate size and ζ-potential was observed precisely when replacing water with the physiological saline solution, while replacing the physiological saline solution with LB broth had a less significant effect. Probably, an increase in the concentration of electrolytes in the physiological solution contributes to an increase in the aggregation of NPs compared to an aqueous medium. Published studies showed that the addition of salts can reduce the colloidal stability of NPs in solution, leading to aggregation due to the formation of a double layer on the surface of each particle [72,73]. Ions absorbed on the particle form the inner layer. Ions with the opposite charge, attracted by the surface charge of the particle, form the outer layer, which shields the original charge and reduces electrostatic interactions between particles [74]. It was shown in the study of the effect of the ionic strength of the solution on the aggregation of gold NPs [72] that at low ionic strength, the NPs formed small aggregates that were stable over a long period of time. When the electrolyte concentration increased, large clusters quickly formed; then, the colloidal suspension became unstable, and precipitation occurred.

### 4.2. Stabilizer Effects

Interesting results were obtained from the analysis of aqueous dispersions obtained using surfactants. In CuO-EE and CuO-CS dispersions, the average aggregate size decreased with the addition of Triton and SDS, while for CuO-CD, an increase in the hydrodynamic diameter was noted. In addition, the ζ-potential indices of CuO-EE and CuO-CS generally increased under the action of stabilizers (except for CuO-EE in the SDS medium), but they decreased in the case of CuO-CD. A similar picture was observed in the physiological saline solution medium. It is known that the addition of surfactants to colloidal suspensions can prevent the agglomeration of colloidal particles [75,76,77].

The observed phenomena can be explained by various molecular mechanisms. For example, the interaction of stabilizer molecules and NPs in a colloidal solution is affected by electrostatic repulsion, which occurs when the NPs in a colloidal solution have the same charge. This prevents their aggregation and promotes solution stability. At the same time, stabilizer molecules can be adsorbed on the surface of NPs, changing their charge. For example, ionic surfactants or polyelectrolytes can enhance the charge, increasing the repulsion. These processes are affected by factors such as the pH of the solution, which affects the ionization of surface groups and the ionic strength of the solution, a high value of which can lead to charge shielding and a decrease in repulsion. Another possible mechanism is steric stabilization, which occurs when long polymer chains of the stabilizer are adsorbed on the surface of NPs. These chains create a physical barrier, preventing particle aggregation. The effectiveness of this barrier is related to the length and flexibility of the polymer chains, the concentration of the polymer in the solution, and the temperature of the solution. In addition, there are known combined cases when NPs are coated with polyelectrolytes that simultaneously create a charge and form a polymer layer [78,79,80].

However, an excessive concentration of colloidal-size additives may induce strong depletion interactions between the larger colloidal particles, resulting in significant flocculation [81]. In our study, CuO-CD NPs had the largest initial sizes. Attractive depletion forces between the larger particles are caused by the exclusion of non-adsorbing colloidal depletants from the gap between the two approaching particles. Then, the concentration difference in depletants between the gap and the bulk solution produces an unbalanced osmotic pressure difference, which yields a net attractive force between the large particles [82,83].

It is noteworthy that the addition of stabilizers reduced the size of CuO-CD particles in LB broth, while the use of SDS resulted in the opposite; it led to an increase in the size of CuO-EE and CuO-CS particles in suspension, without a significant change in ζ-potential.

The study of the antibacterial properties of aqueous CuO dispersions (100 mg L^−1^) after 12 h of exposure showed that the greatest suppression of culture growth occurred in a medium without stabilizers, where the OD600 indicator decreased for all tested NPs. The lowest indicator was in the CuO-EE variant—30% lower than the control values. It is worth noting that it was for this version of the dispersion for which the highest positive ζ-potential indicator was also observed. According to [84,85], NPs with a positive charge can have a higher bactericidal effect. The bacterial membrane carries a negative charge mainly due to phosphates and carboxylates contained in lipopolysaccharides [86]. Published studies showed that the accumulation of positive charge on the surface of NPs provides electrostatic interaction with the negatively charged membrane [87,88]. Subsequently, NPs can accumulate inside the cell with the formation of ROS, which, in turn, disrupts protein efflux and the work of ATPasic pumps, disrupting the integrity of membranes and inhibiting cellular protein synthesis, which will ultimately lead to the death of bacterial cells [86]. The recent studies show that the cytotoxicity of Cu-containing NPs may be associated with the ability of Cu to replace or bind to native cofactors in metalloproteins. Intracellular Cu accumulation promotes abnormal metal formation, which is mainly associated with the iron–sulfur cluster protein and its assembly process. In particular, Cu accumulated in bacterial cells mainly exists in the form of highly toxic Cu^+^, which interacts with thiolate or inorganic sulfur ligands of solvent-exposed dehydratase and replaces the iron atom, rapidly inactivating Fe/S cluster dehydratases and causing cellular dysfunction [32]. It should be noted that this mechanism of toxicity is unlikely in our study, as we used CuO NPs; accordingly, the release of Cu ions from the surface will be insignificant, which is confirmed by the data on the concentration of Cu ions in the tested dispersions (Supplementary Materials, Table S4).

As mentioned above, the addition of Triton X-100 and SDS changed the charge of CuO-EE in the solution to negative, thereby removing the antibacterial effect of these NPs. In the case of CuO-CD and CuO-CS, culture growth was also inhibited regardless of the stabilizers. Replacing water with the physiological saline solution reduced the negative effect of NPs on bacteria. At the same time, interesting results were obtained for CuO-EE; despite the fairly high positive charge (24.5 mV) of particles in the medium with Triton X-100, this dispersion did not have a negative effect on microorganisms, and moreover, there was a stimulation of culture growth in this case. The stabilizers used did not affect the physiological saline solution.

We have previously shown a decrease in the antibacterial activity of TiS_3_ nanostructures [60] in a physiological saline solution medium compared to aqueous dispersions, as well as when replacing nutrient broth with the physiological saline solution for graphene oxide (GO) [29] and reduced GO (rGO) [31]. In the LB broth medium, dispersions without a stabilizer and dispersions with Triton X-100 did not have a toxic effect on bacteria, but when using SDS, the culture density decreased by 1.5 times when exposed to CuO-CD, by 1.8 times when adding CuO-EE, and by 1.3 times in the case of CuO-CS. In the LB broth, both surfactants had a toxic effect on microorganisms. It is possible that the observed effects are associated with the intrinsic biological action of the stabilizers used. In contrast to Triton X-100, which exhibits limited antibacterial activity [43], SDS exhibits antibacterial and cytotoxic effects associated with its detergent effect on cellular differentiation and on barrier lipids in the membrane [89]. In addition, it is known that ionic and nonionic surfactants have different effects on the antibacterial properties of NPs in colloidal systems. For example, when studying the influence of various surfactants on the effects of nanosilver on *E. coli* bacteria, a predominant contribution of such particle surface properties as charge and hydrophobicity was shown, while suspension stability was of lesser importance [90].

### 4.3. Sise and Shape Effects of CuO NPs

It is important to note that the experiment failed to reveal the dependence of antibacterial effects on particle size. Moreover, in some cases, CuO-EE electroexplosive particles, which, according to SEM data, have minimal sizes, had the least antibacterial effect. However, CuO-CS NPs, which have a rod-shaped form, showed relatively high antibacterial efficiency. This is consistent with the results of the work by [91], where the important role of the shape of CuO NPs in the formation of their toxic properties is shown. Similar results were obtained in [38]. Rod-shaped Fe_2_O_3_ nanoparticles also induced higher levels of necrosis, membrane damage, and ROS generation in mouse macrophage cells (RAW 264.7) compared to spherical Fe_2_O_3_ nanoparticles [92].

### 4.4. Concentration Effects of CuO NPs

The antibacterial effects of the CuO NP concentration were multidirectional and nonlinear. In general, aqueous dispersions of CuO NPs in the minimum concentration (1 mg L^−1^) had the highest toxicity, regardless of the presence of a stabilizer. Similar nonlinear effects for Cu NPs were noted by us earlier in [51]. Nonlinear dose effects have been identified in several previous nanotoxicological studies. The authors associate them with stepwise aggregation processes in colloidal solutions [93], the abrupt nature of adaptation of biological objects [94], or signaling effects of NPs. They can also be associated with non-monotonic characteristics of such mechanisms of NP toxicity as ROS generation or penetration through the cell membrane. In [95], the concentration–effect curves of soybean antioxidant biomarker activity were linear for 25 nm CuO NPs and Cu^2+^, whereas for 50 and 250 nm CuO NPs, the effects were nonlinear. The studies showed that NPs can cause toxicity at low concentrations by disrupting various cellular functions and altering mechanobiological behaviors [96]. These mechanisms require further study.

In the physiological saline solution medium, the NPs had virtually no effect on the microorganisms, with the exception of dispersions obtained with SDS. Moreover, despite the absence of bactericidal action of the pure stabilizer, in its presence, CuO-EE and CuO-CS NPs at 1 mg L^−1^, as well as CuO-CD and CuO-EE at 10 mg L^−1^, inhibited culture growth. Only in the case of CuO-CS did the OD value decreased at the maximum concentration, regardless of the use of surfactant. In the case of LB broth without stabilizers, the inhibition of culture growth was also noted for all variants of NPs at 1 mg L^−1^; the minimum OD values were recorded when adding CuO-CS to the medium. It is noteworthy that an increase in the CuO-CS concentration to 10 mg L^−1^ leveled the negative effect of the NPs, and a stimulating effect was noted at 100 mg L^−1^. CuO-CD and CuO-EE had no significant effect on bacteria at 10 and 100 mg L^−1^. CuO-CD and CuO-CS dispersions obtained using Triton X-100 inhibited cell growth at 1 mg L^−1^. The cultivation of *E. coli* with 10 mg L^−1^ CuO-CS also resulted in a significant decrease in OD. As in the case of the physiological saline solution, in the LB broth medium, the most toxic CuO NP dispersions were obtained using SDS. The lowest OD values (an average 1.8-fold decrease relative to the control) were noted for all types of NPs at 1 and 10 mg L^−1^.

Thus, aqueous CuO NP dispersions containing 1 mg L^−1^ in water and LB broth, as well as dispersions prepared using SDS, had the greatest antibacterial properties. The qualitative results of the microbiological experiments that we obtained are summarized in Table 1.

Table 1 shows that there is no clear relationship between the size of CuO NPs and their antibacterial properties, but a significant effect of the dispersion medium on toxicity towards *E. coli* bacteria is revealed, which is a very important factor in the development of antibacterial coatings containing NPs.

An important practical result of our work was the understanding that the antibacterial properties of CuO NPs can vary from zero to maximum solely due to the properties of the environment. Of course, the identification of specific mechanisms of the antibacterial action of CuO NPs in light of the obtained data on the leading role of the dispersion medium requires further research, as well as further mechanistic studies of the interaction of CuO NPs with prokaryotic and eukaryotic cells. In addition, the method of measuring OD600 used in our study, although it is the most common method for assessing the number of cells in a liquid suspension due to its high productivity, easy automation, simplicity, and low cost, does not allow for a high-precision determination of the number of living and dead cells or for identification of toxicity mechanisms. Alternative measurements of cell count, such as microscopy, colony-forming units, and others, may offer this and other advantages, including more accurate assessments of viability and independence from cell states, inclusion body formation, protein expression, or filamentous growth [97]. However, they lack many of the valuable properties provided by the method of measuring OD600 used in our study. In this regard, further studies are needed using other methods, such as flow cytometry and microscopy, which would not only allow for the determination of the number of cells but also the mechanisms of antibacterial action.

Finally, it is worth noting that none of the concentrations studied (1, 10, and 100 mg/L) showed complete inhibition of microorganisms. Moreover, in our earlier study [98], no complete inhibition of *E. coli* growth was observed at a concentration of 1 mg/mL. Thus, nonlinear effects and the lack of complete growth inhibition even at the highest concentration do not allow us to correctly calculate the minimum inhibitory concentration (MIC). Meanwhile, the available literature data on the MIC of CuO NPs for *E. coli* are contradictory. Thus, Khairy et al. [99] showed that the MIC for multidrug-resistant *E. coli* was 62.5 µg/mL for CuO NPs biosynthesized using ethanolic Neem extract and 125 µg/mL for CuO NPs biosynthesized using ethanolic jojoba extract. The MIC for CuO NPs biosynthesized using *Athrixia phylicoides* DC was 2.5 mg/mL [100]. The MIC for CuO NPs synthesized via a mechanochemical method using two different Cu-containing precursors was 3.75 µg/mL. Thus, it can be concluded that the antibacterial properties of NPs are significantly influenced by the physicochemical properties of their surface associated with the synthesis method.

## 5. Conclusions

Thus, the chemical nature of the medium, as well as the presence and chemical nature of the stabilizer had the greatest influence on the optical density (OD) value. The maximum antibacterial effect for all types of copper (II) oxide nanoparticles (CuO NPs) was found in water without a stabilizer, as well as in LB broth with the sodium dodecyl sulfate (SDS) stabilizer. In saline, the antibacterial effects were minimal, and in some cases, culture growth was observed. SDS increased the antibacterial effects of NPs in broth and saline but decreased them in water. Finally, among the types of CuO NPs, commercial sample CuO-CS was the most toxic (this is probably due to their rod-shaped morphology and small diameter). Interestingly, the smallest CuO-EE particles obtained by electrical explosion not only turned out to be the least bactericidal but also exhibited stimulating properties as the negative charge of the surface increased and the size of aggregates in colloids decreased. At the same time, the concentration of NPs and the effects of aggregation in the colloidal systems we studied did not have a decisive effect on their antibacterial properties, which, given the numerous evidence to the contrary, was unexpected.

The results obtained can be used in the creation of antibacterial coatings and preparations based on CuO NPs to achieve their maximum efficiency, taking into account the expected conditions of use.

## Figures and Tables

**Table 1:** Effects of different types of CuO NPs, their concentrations, stabilizers, and types of media on the value of the OD index (“−” decrease, “+” increase, and “0” no effect).

CuO NPs, Concentrations, mg L^−1^	Stabilizers and Types of Media								
	No Stabilizer			Triton X-100			SDS		
	Water	Physiological Saline Solution	LB Broth	Water	Physiological Saline Solution	LB Broth	Water	Physiological Saline Solution	LB Broth
CuO-CD									
1	−	0	−	−	+	−	−	0	−
10	−	0	0	−	+	0	0	−	−
100	−	0	0	−	+	0	−	0	−
CuO-EE									
1	−	0	−	−	0	0	−	−	−
10	−	0	0	0	0	0	0	−	−
100	−	0	0	-	0	0	0	0	−
CuO-CS									
1	−	0	−	−	0	−	−	−	−
10	−	0	0	−	0	−	0	0	−
100	−	−	+	−	−	0	0	−	−

## Data Availability

Data are contained within the article.

## Supplementary Materials

The following supporting information can be downloaded at https://www.mdpi.com/article/10.3390/nano15060469/s1, Figure S1. Effect of 100 mg L^−1^ CuO NPs on *E. coli* bacteria in different media: (a) distilled water, (b) physiological saline solution, and (c) LB broth; Table S1. Effects of different types of CuO NPs, their concentrations, stabilizers, and types of media on the luminescence intensity of *E. coli*; Table S2. Effects of Triton X-100 in different media on the luminescence intensity of *E. coli*; Table S3. Effects of SDS in different media on the luminescence intensity of *E. coli*.; Table S4. Cu^2+^ concentration measurements results.
