# Supplementary material for: The Composition of the Dispersion Medium Determines the Antibacterial Properties of Copper (II) Oxide Nanoparticles Against Escherichia coli Bacteria
Source: Nanomaterials (Basel). 2025 Mar 20;15(6):469. doi: 10.3390/nano15060469 (PMC11944915; doi:10.3390/nano15060469)
Supplement: Supplementary file 1 [file nanomaterials-15-00469-s001.zip › nanomaterials-3478063-supplementary.pdf]

nanomaterials

MDPI

Supplementary materials

The composition of the dispersion medium determines the antibacterial properties of copper (II) oxide nanoparticles against *E. coli* bacteria

Olga V. Zakharova [1,2,*], Alexander A. Gusev [1,2], Peter A. Baranchikov [1,2], Svetlana P. Chebotaryova [1,2], Svetlana S. Razlivalova [1,2], Elina Y. Koiava [1,2], Anna A. Kataranova [1,2], Gregory V. Grigoriev [1,2], Nataliya S. Strekalova [1], Konstantin V. Krutovsky [3,4,5,6,*]

1 Scientific and Educational Center for Environmental Science and Biotechnology, Derzhavin Tambov State University, 392020 Tambov, Russia; olgazakharova1@mail.ru (O.V.Z.); nanosecurity@mail.ru (A.A.G.); petrovi4-98@yandex.ru (P.A.B.); sweta-chebotarjova@yandex.ru (S.P.C.); razlivalova8@yandex.ru (S.S.R.); e.koiava.e.02@mail.ru (E.Y.K.); akataranova@bk.ru (A.A.K.) bboykick@outlook.com (G.V.G.); kotova-ns@yandex.ru (N.S.S.)
2 Department of Functional Nanosystems and High-Temperature Materials, National University of Science and Technology «MISIS», 119991 Moscow, Russia
3 Department of Forest Genetics and Forest Tree Breeding, Faculty of Forest Sciences and Forest Ecology, Georg-August University of Göttingen, Büsgenweg 2, 37077 Göttingen, Germany; konstantin.krutovsky@forst.uni-goettingen.de (K.V.K.)
4 Laboratory of Population Genetics, N.I. Vavilov Institute of General Genetics, Russian Academy of Sciences, Gubkin Str. 3, 119333 Moscow, Russia
5 Genome Research and Education Center, Laboratory of Forest Genomics, Department of Genomics and Bioinformatics, Institute of Fundamental Biology and Biotechnology, Siberian Federal University, 660036 Krasnoyarsk, Russia
6 Scientific and Methodological Center, G.F. Morozov Voronezh State University of Forestry and Technologies, 8 Timiryazeva Str., 394036 Voronezh, Russia
* Correspondence: olgazakharova1@mail.ru (O.V.Z.); konstantin.krutovsky@forst.uni-goettingen.de (K.V.K.)

Additional Methods and Results

Antibacterial properties

To study the toxicity of copper nanoparticle (Cu NP) solutions, a bioluminescent technique was used, which is used for microbiological assessment of the impact of nanomaterials on microbiomes [1-3]. The method is based on determining the change in the bioluminescence intensity of the genetically engineered strain of *E. coli* K12 TG1 bacteria when exposed to NPs present in the analyzed sample compared to the control. The criterion for toxic action is the change in the bioluminescence intensity of the test object in the studied sample compared to the control, which does not contain toxic substances. A decrease in the bioluminescence intensity is proportional to the toxic effect.

The toxic effect of the studied nanomaterial sample on bacteria is determined by the inhibition of their bioluminescence over a 12 h exposure period. The quantitative assessment of the test reaction parameter is expressed as the toxicity index (T), a dimensionless value calculated using the formula: $T = 100 \times (I_o - I)/I_o$, where I_o and I are the luminescence intensity in the control and the experiment, respectively, at a fixed exposure time of the studied sample with the test object.

The method provides three threshold levels of T degree:
1) acceptable: T = 0-20;
2) average degree: T = 21-50;
3) high degree: T > 50.

Negative values of T are considered as absence of toxicity.

When determining T, parallel measurements of control and test samples were carried out. For greater reliability of the data, the measurements were repeated five times. The result of the toxicological analysis is presented as arithmetic mean of T, that is $\bar{T} \pm \sigma$, where $\bar{T} = \frac{1}{n}\sum_{i=1}^{n} T_i$ for n repetitions, and σ – standard deviation, which is determined by generally accepted formula $\sigma = \sqrt{\frac{\sum_{i=1}^{n}(\bar{T}-T_i)^2}{n-1}}$, where T_i – ith result of determining T, and n is the number of repetitions.

The measurements were carried out on a portable Biotox-10 device (Nera S, Moscow, Russia).

Quality control of the toxicity assessment was carried out by determining the sensitivity of the test organism used to the model "reference" toxicant: zinc sulfate heptahydrate ($ZnSO_4 \cdot 7H_2O$) (Merck, Darmstadt, Germany), dissolved in

distilled water (pH 6.8-7.4). The concentration of the model toxicant, under the action of which after 30 minutes of exposure the intensity of bioluminescence is inhibited by at least 50%, should be no more than 4.4 mg mL^{-1}.

Results of the analysis of antibacterial properties of suspensions with a concentration 100 mg L^{-1} presented in Tables S1-S3 and Figure S1. The diagrams in Figure S1 demonstrate that the data obtained by the bioluminescent method are comparable with the data of spectrophotometric analysis (see Figure 1 in the main text).

Table S1. Effects of different types of CuO NPs, their concentrations, stabilizers, and types of media on the luminescence intensity of *E. coli*.

Nanoparticle	Luminescence, pulses / second		Average toxicity index, units	Luminescence, pulses / second		Average toxicity index, units	Luminescence, pulses / second		Average toxicity index, units
	control	CuO-CD		control	CuO-EE		control	CuO-CS	
Water dispersions									
CuO	6035	5210	14.7	6108	4322	30.7	6017	5155	15.0
	6059	5214		6143	4216		6100	5143	
	6114	5104		6113	4316		6039	5155	
	6023	5162		6114	4155		6077	5066	
	6000	5101		6091	4186		6014	5187	
CuO+Triton	6132	5133	16.5	6031	6206	-2.8	6010	5199	15.2
	6100	5079		6096	6226		6045	5122	
	6072	5030		6010	6282		6074	5181	
	6063	5069		6132	6288		6129	5068	
	6066	5080		6145	6266		6048	5129	
CuO+SDS	6140	4260	29.2	6073	6768	-9.4	6037	5271	12.9
	6013	4393		6098	6695		6072	5173	
	6088	4270		6084	6650		6023	5296	
	6041	4222		6095	6631		6072	5374	
	6073	4344		6131	6617		6067	5239	
Physiological solution									
CuO	6141	6158	-1.2	6115	5909	1.7	6139	5637	3.9
	6148	6212		6098	5918		6086	5636	
	6085	6096		6004	5938		6096	5973	
	6016	6173		6147	6095		6062	6071	
	6075	6187		6058	6038		6115	5968	
CuO+Triton	6130	6170	0.9	6126	6507	-6.2	6094	5774	3.1
	6137	6008		6051	6404		6101	5993	
	6088	5852		6126	6562		6134	5832	
	6076	6038		6138	6392		6092	5935	
	6097	6169		6098	6584		6144	6080	
CuO+SDS	6035	6242	-2.1	6035	7120	-12.5	6013	5294	12.4
	6084	6325		6039	6998		6054	5503	
	6021	6168		6136	6448		6094	5179	
	6042	6059		6055	7087		6001	5244	
	6030	6069		6038	6428		6055	5248	

Nanoparticle	Luminescence, pulses / second		Average toxicity index, units	Luminescence, pulses / second		Average toxicity index, units	Luminescence, pulses / second		Average toxicity index, units
	control	CuO-CD		control	CuO-EE		control	CuO-CS	
LB broth									
CuO	6082	6180	-2.5	6133	6189	-2.1	6138	6074	-1.7
	6066	6132		6000	6052		6014	6239	
	6054	6395		6009	5967		6083	6175	
	6042	6054		6056	6351		6097	6151	
	6054	6287		6118	6405		6026	6235	
CuO+Triton	6112	6062	0.9	6116	6332	-3.2	6011	6033	0.3
	6138	6093		6037	6059		6100	6037	
	6132	6181		6038	6410		6004	6042	
	6133	6019		6134	6058		6038	5983	
	6014	5880		6094	6543		6074	6044	
CuO+SDS	6134	4321	29.6	6100	3824	38.1	6093	4381	29.7
	6113	4234		6039	3740		6001	4255	
	6066	4292		6067	3712		6102	4395	
	6060	4377		6049	3831		6104	4205	
	6147	4255		6077	3661		6113	4151	

Table S2. Effects of Triton X-100 in different media on the luminescence intensity of *E. coli*.

Water			Physiological solution			LB broth		
Luminescence, pulses / second		Average toxicity index, units	Luminescence, pulses / second		Average toxicity index, units	Luminescence, pulses / second		Average toxicity index, units
control	Triton X-100		control	Triton X-100		control	Triton X-100	
6002	4280	28.4	6119	6092	-0.4	6054	5010	17.7
6068	4301		6032	6121		6072	4918	
6099	4405		6086	6073		6072	5090	
6028	4415		6014	6039		6092	4969	
6069	4353		6059	6121		6143	5042	

Table S3. Effects of SDS in different media on the luminescence intensity of *E. coli*.

Water			Physiological solution			LB broth		
Luminescence, pulses / second		Average toxicity index, units	Luminescence, pulses / second		Average toxicity index, units	Luminescence, pulses / second		Average toxicity index, units
control	SDS		control	SDS		control	SDS	
6062	6247	0	6004	6044	0	6109	3003	49.9
6019	6135		6055	6121		6030	3072	
6028	6006		6091	6024		6042	3124	
6059	6021		6128	6081		6043	2952	
6022	6031		6006	6026		6061	3020	

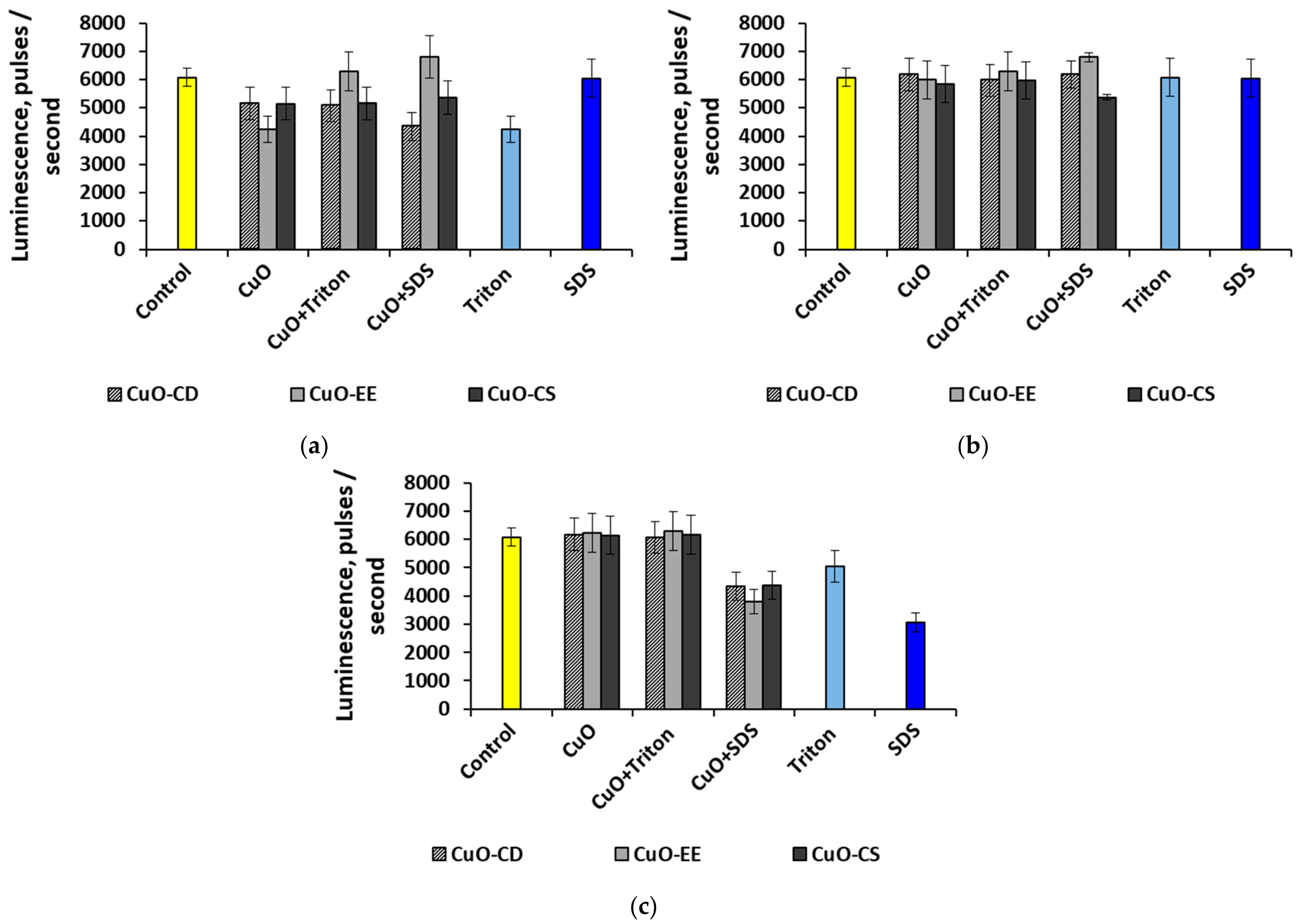

Figure S1. Effect of 100 mg L^{-1} CuO NPs on *E. coli* bacteria in different media: (a) distilled water, (b) physiological saline solution, (c) LB broth.

Cu^{2+} concentration measurements

To assess the effect of Cu ions on bacteria, measurements of Cu^{2+} concentrations were carried out for dispersions containing 100 mg L^{-1} CuO NPs using the liquid analyzer "Expert-001" (Econix Expert, Moscow, Russian Federation). The results of the analysis are presented in Table S4.

Table S4. Cu^{2+} concentration measurements results.

Treatment variant	5 min after preparation		12 h after preparation	
	mg L^{-1}	mM	mg L^{-1}	mM
CuO-CS in water	0.02	0.0004	0.02	0.0004
CuO-CD in water	0.03	0.0004	0.05	0.0007
CuO-EE in water	0.03	0.0004	0.02	0.0003
CuO-CS + Triton in water	0.03	0.0005	0.03	0.0006
CuO-CD + Triton in water	0.04	0.0005	0.04	0.0007
CuO-EE + Triton in water	0.03	0.0004	0.03	0.0005
CuO-CS + SDS in water	0.03	0.0005	0.03	0.0005
CuO-CD + SDS in water	0.04	0.0005	0.04	0.0005
CuO-EE + SDS in water	0.02	0.0004	0.02	0.0004

Treatment variant	5 min after preparation		12 h after preparation	
	mg L^{-1}	mM	mg L^{-1}	mM
CuO-CS in physiological saline solution	0.03	0.0004	0.03	0.0005
CuO-CD in physiological saline solution	0.03	0.0004	0.03	0.0006
CuO-EE in physiological saline solution	0.03	0.0004	0.02	0.0005
CuO-CS + Triton in physiological saline solution	0.03	0.0005	0.03	0.0005
CuO-CD + Triton in physiological saline solution	0.03	0.0005	0.02	0.0007
CuO-EE + Triton in physiological saline solution	0.03	0.0005	0.03	0.0007
CuO-CS + SDS in physiological saline solution	0.04	0.0004	0.03	0.0005
CuO-CD + SDS in physiological saline solution	0.03	0.0004	0.03	0.0005
CuO-EE + SDS in physiological saline solution	0.03	0.0005	0.03	0.0006
CuO-CS in LB broth	0.08	0.0014	0.08	0.0013
CuO-CD in LB broth	0.1	0.0015	0.09	0.0014
CuO-EE in LB broth	0.1	0.0014	0.08	0.0013
CuO-CS + Triton in LB broth	0.08	0.0013	0.08	0.0013
CuO-CD + Triton in LB broth	0.09	0.0014	0.08	0.0014
CuO-EE + Triton in LB broth	0.09	0.0014	0.08	0.0013
CuO-CS + SDS in LB broth	0.1	0.0014	0.08	0.0013
CuO-CD + SDS in LB broth	0.02	0.0004	0.02	0.0004
CuO-EE + SDS in LB broth	0.03	0.0004	0.05	0.0007

References

1. Zakharova, O.V.; Belova, V.V.; Baranchikov, P.A.; Kostyakova, A.A.; Muratov, D.S.; Grigoriev, G.V.; Chebotaryova, S.P.; Kuznetsov, D.V.; Gusev, A.A. The Conditions Matter: The Toxicity of Titanium Trisulfide Nanoribbons to Bacteria *E. coli* Changes Dramatically Depending on the Chemical Environment and the Storage Time. *Int. J. Mol. Sci.* **2023**, *24*(9), 8299. https://doi.org/10.3390/ijms24098299
2. Zakharova, O.V.; Gusev, A.A.; Abourahma, J.; Vorobeva, N.S.; Sokolov, D.V.; Muratov, D.S.; Kuznetsov, D.V.; Sinitskii, A. Nanotoxicity of ZrS_3 Probed in a Bioluminescence Test on *E. coli* Bacteria: The Effect of Evolving H_2S. *Nanomaterials* **2020**, *10*(7), 1401. https://doi.org/10.3390/nano10071401
3. Zakharova, O.V.; Godymchuk, A.Y.; Gusev, A.A.; Gulchenko, S.I.; Vasyukova, I.A.; Kuznetsov, D.V. Considerable Variation of Antibacterial Activity of Cu Nanoparticles Suspensions Depending on the Storage Time, Dispersive Medium, and Particle Sizes. *Biomed Res. Int.* **2015**, *2015*, 412530. https://doi.org/10.1155/2015/412530
